# Behaviour change interventions to influence antimicrobial prescribing: a cross-sectional analysis of reports from UK state-of-the-art scientific conferences

**DOI:** 10.1186/s13756-017-0170-7

**Published:** 2017-01-13

**Authors:** T. M. Rawson, L. S. P. Moore, A. M. Tivey, A. Tsao, M. Gilchrist, E. Charani, A. H. Holmes

**Affiliations:** 1 National Institute for Health Research Health Protection Research Unit in Healthcare Associated Infections and Antimicrobial Resistance, Imperial College London, Hammersmith Campus, Du Cane Road, London, W12 0NN UK; 2 Imperial College School of Medicine, Imperial College London, South Kensington Campus, London, SW7 2AZ UK; 3 Imperial College Healthcare NHS Trust, Hammersmith Hospital, Du Cane Road, London, W12 0HS UK

**Keywords:** Antimicrobial Resistance, Stewardship, Quality improvement, Cross-specialty, Infection

## Abstract

**Background:**

To improve the quality of antimicrobial stewardship (AMS) interventions the application of behavioural sciences supported by multidisciplinary collaboration has been recommended. We analysed major UK scientific research conferences to investigate AMS behaviour change intervention reporting.

**Methods:**

Leading UK 2015 scientific conference abstracts for 30 clinical specialties were identified and interrogated. All AMS and/or antimicrobial resistance(AMR) abstracts were identified using validated search criteria. Abstracts were independently reviewed by four researchers with reported behavioural interventions classified using a behaviour change taxonomy.

**Results:**

Conferences ran for 110 days with >57,000 delegates. 311/12,313(2.5%) AMS-AMR abstracts (oral and poster) were identified. 118/311(40%) were presented at the UK’s infectious diseases/microbiology conference. 56/311(18%) AMS-AMR abstracts described behaviour change interventions. These were identified across 12/30(40%) conferences. The commonest abstract reporting behaviour change interventions were quality improvement projects [44/56 (79%)]. In total 71 unique behaviour change functions were identified. Policy categories; “guidelines” (16/71) and “service provision” (11/71) were the most frequently reported. Intervention functions; “education” (6/71), “persuasion” (7/71), and “enablement” (9/71) were also common. Only infection and primary care conferences reported studies that contained multiple behaviour change interventions. The remaining 10 specialties tended to report a narrow range of interventions focusing on “guidelines” and “enablement”.

**Conclusion:**

Despite the benefits of behaviour change interventions on antimicrobial prescribing, very few AMS-AMR studies reported implementing them in 2015. AMS interventions must focus on promoting behaviour change towards antimicrobial prescribing. Greater focus must be placed on non-infection specialties to engage with the issue of behaviour change towards antimicrobial use.

**Electronic supplementary material:**

The online version of this article (doi:10.1186/s13756-017-0170-7) contains supplementary material, which is available to authorized users.

## Background

In the United Kingdom (UK), about one third of all hospital inpatients receive antimicrobials during their admission with a significant proportion of these identified as inappropriate [1–3]. This accounts for a large amount of unnecessary antimicrobial exposure. Antimicrobial resistance (AMR) is a leading patient safety issue that requires urgent interventions to curb its exponential growth. One target of interventions to address the problem of AMR is the promotion of the appropriate use of antimicrobials in humans, which is thought to be a leading driver for the growth of AMR [4].

To address this and promote the appropriate use of antimicrobial agents a number of national and international antimicrobial stewardship (AMS) initiatives have been implemented [5–8]. A key facet of these interventions targets improving and sustaining individual prescribing behaviours. Implementation of AMS programmes have been demonstrated to reduce rates of AMR and improve health and economic outcomes [9–11]. However, despite these positive steps forward, several challenges appear to remain in promoting the sustainable use of antimicrobials across clinical practice [12].

Firstly, there is a growing body of evidence to describe the cultural and social factors that influence antimicrobial prescribing across healthcare settings as well as qualitative data that supports the role of behaviour change interventions in improving antimicrobial prescribing [13–16]. Despite this, very little evidence exists to describe the current landscape of behaviour change interventions being implemented within this field [12, 13, 15–20]. Secondly, despite evidence to support engagement of infection specialists with the AMS-AMR agenda, there appears to be poorer engagement across other clinical specialties in terms of formal training and awareness at state-of-the-art scientific conferences [21–23]. Finally, although there are described frameworks and taxonomy’s available from which to begin mapping behaviour change methods [24, 25], very little data is currently available to describe the appropriateness of these specifically for AMS interventions.

In this cross-sectional study we aimed to explore antimicrobial stewardship interventions reported at major cross specialty UK state-of-the-art scientific conferences in 2015, which contained behaviour change interventions. We aimed to determine the number and type of behaviour change interventions reported by different specialties and compare these to currently available behaviour change taxonomies to identify potential gaps and highlight potential targets for future interventions.

## Methods

### Abstract identification & screening

All major medical specialties recognised by the Royal College of Physicians, London, UK, were identified and included alongside major surgical specialties identified by the intercollegiate surgical curriculum programme. Psychiatric, paediatric, and obstetrics and gynaecology specialties were also included. UK specialists (specialist trainees or consultants) in each of the defined fields were consulted by email to determine the largest clinical scientific/research conference within the UK in 2015. Two specialists from each field, who were based in the North West London area were contacted for their opinions. Where there was disagreement, the authors opted for the conference with the largest attendance. Educational, continuing professional development and sub-specialty conferences were not considered for inclusion given their often focused agendas, which may have biased our findings.

Each major conference per specialty was identified and abstract booklets extracted and interrogated. Conference characteristics collated included; location, conference dates, estimated attendance and total number of abstracts accepted (either as oral, poster or publication only). Accepted conference abstracts (invited, oral, poster and publication only) were then identified and interrogated using a previously validated search criterion to identify all abstracts relating to AMS and AMR. [21, 22] All identified oral, poster, or published only abstracts from the search were then anonymously reviewed by two out of three authors (TMR, AMT, & AT). Abstracts were included if they were deemed to be describing an aspect of AMS [26] or AMR [27] in terms of direct effect on patients. In vitro studies with no translational benefit to individual patients were excluded. For the purpose of our investigation we focused on bacterial resistance and stewardship, abstracts relating solely to antiviral, antifungal, antiprotozoal or antimycobacterial resistance were excluded. This focus was selected given that anti-bacterial agents make up over 93% of all antimicrobials prescribed for systemic use [28]. Furthermore, the large variation in prescribing of other antimicrobial classes across different specialties may have influenced our results. When there was disparity between the opinions of reviewers’ a fourth independent reviewer (LSPM) was consulted to reach consensus.

### Characterising behaviour change interventions

Once all AMS-AMR abstracts had been identified the rates of AMS-AMR coverage between specialty conferences was assessed. Abstracts were then re-read by at least two of four researchers (TMR, AMT, AT, & LSPM) and categorised into types of intervention reported in the abstracts. To categorise the types of interventions reported a modified version of intervention and policy framework definitions provided by Michie and colleagues for the construction of their behaviour change wheel were used (Additional file 1: Table S1) [25]. In the original behaviour change wheel, three layers (policy, intervention, and behaviour systems are described). Within the classification used in this study, behaviour systems were not included (capability, opportunity, motivation, and behaviour; COM-B) as reported interventions were focused on the two levels of the framework above this, which aim to directly influence COM-B [25]. Researchers attempted, where possible, to categorise reported behaviour change interventions into one or more of the sixteen functions (split into policies and interventions) described within this framework. Although the framework is designed to provide flexibility and accommodate multiple interventions/policy combinations, researchers attempted to strictly categorise reported interventions into the fewest number of categories possible. When there was discrepancy the group discussed these issues until consensus was reached. Descriptive statistics was performed in SPSS 22.0 (IBM, Chicago, IL) with Chi-squared with Yates correction. Ethics approval was not required for this observational study.

## Results

### AMS-AMR coverage at UK state-of-the-art scientific conferences in 2015

**Fig. 1:**
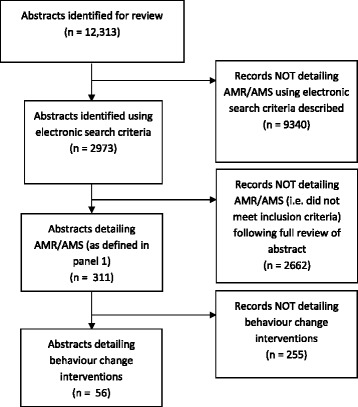
Selection method to identify antimicrobial stewardship/antimicrobial resistance abstracts among state-of-the-art conferences in 2015

Thirty specialty state-of-the-art scientific conferences abstract booklets were identified and extracted for analysis. These conferences ran over >110 days with >57,000 delegates estimated to of attended them in 2015 (Table 1). In total, 12,313 abstracts were extracted for analysis with 311 (2.5%) identified as related to AMS-AMR (Fig. 1). Of these, 118/311 (38%) were presented at the UK’s infectious diseases/microbiology conference [29]. This made up 38% (144/375) of all conference abstracts reported at this conference. Genitourinary medicine [30] had the second highest coverage with 9% (26/299), orthopaedics [31] third and plastic surgery [32] fourth with 8% of abstracts related to AMS-AMR each (8/96 & 6/78, respectively). All other specialty’s had <5% AMS-AMR coverage with neurology [33], emergency medicine [34], psychiatry [35], geriatrics [36], and endocrinology [37] not having any AMS-AMR related coverage at their 2015 conferences. Compared to published data on conference coverage in the UK in 2014 [21] there was no significant difference in the level of AMS-AMR reporting (311/12,313, 2.5%, in 2015 & 221/7843, 2.8%, in 2014; *p* = 0.22). Infection/microbiology had a significantly larger proportion of AMS-AMR abstracts compared to all other specialties reviewed within this study (*p* < 0.01).

**Table 1:** UK state-of-the-art scientific conference summary

Speciality	City	Date commenced	No Days	No delegates	No abstracts accepted
Anaesthetics [44]	Edinburgh	23/09/2015	3	800	161
Breast Surgery [45]	Bournemouth	15/06/2015	2	870	221
Cardiology [46]	Manchester	08/06/2015	3	2448	235
Dermatology [47]	Manchester	06/07/2015	4	1200	372
Emergency Medicine [34]	Manchester	28/09/2015	3	650	69
Endocrinology [37]	Edinburgh	02/11/2015	3	1000	526
Gastroenterology [48]	London	22/06/2015	4	4500	1240
Primary Care [38]	Glasgow	01/10/2015	3	1600	450
General Surgery [49]	Manchester	22/04/2015	3	1500	1065
Surgery (ASiT) [50]	Glasgow	27/02/2015	3	700	602
Genitourinary Medicine [30]	Glasgow	01/06/2015	3	500	299
Geriatrics [36]	Brighton	14/10/2015	3	500	76
Haematology [51]	Edinburgh	20/04/2015	3	1000	257
Infection/Microbiology [29]	Glasgow	21/11/2015	3	1000	375
Intensive Care [52]	London	07/12/2015	3	1250	154
Nephrology [43]	London	28/05/2015	4	8190	1945
Neuro surgery [53]	York	09/09/2015	3	200	139
Neurology [33]	Harrogate	20/05/2015	3	600	194
Obstetrics & Gynaecology [54]	Brisbane	12/04/2015	4	2300	770
Ophthalmology [55]	Liverpool	18/05/2015	4	1700	228
Orthopaedics [31]	Liverpool	15/09/2015	4	1600	96
Paediatric surgery [56]	Cardiff	22/07/2015	3	346	83
Paediatrics [57]	Birmingham	28/04/2015	3	2000	546
Plastic surgery [32]	Birmingham	25/11/2015	3	400	78
Psychiatry [35]	Birmingham	29/06/2015	4	2500	79
Respiratory [58]	London	02/12/2015	3	2200	460
Rheumatology [59]	Manchester	28/04/2015	3	2000	677
Transplant surgery [60]	Bournemouth	11/03/2015	3	700	382
Urology [61]	Manchester	15/06/2015	4	1200	161
Vascular surgery [62]	Bournemouth	11/11/2015	3	800	373

### Reported behaviour change interventions for antimicrobial prescribing

Of the 311 AMS-AMR abstracts identified 56 (18%) described behaviour change interventions (Table 2). Of these, 28/56 (50%) were reported at the infection/microbiology conference with general surgery conferences reporting the second largest proportion with 7/56 (13%). In total, behaviour change interventions were reported across 12/30 (40%) specialty state-of-the-art conferences with infection/microbiology reporting a significantly greater amount that all other conferences (*p* < 0.01). The commonest abstracts reporting behaviour change interventions were quality improvement projects accounting for 44/56 (79%) of all reported behaviour change interventions. However, this represented a minority of all AMS-AMR quality improvement projects identified with 80/124 (65%) either not reporting any intervention or not reporting a specific behaviour change intervention. The remainder of behaviour change interventions included were found to be reported within observational studies (12/56; 21%). This also represented a minority of observational studies reporting AMS-AMR topics across clinical specialties (12/54; 22%).

**Table 2:** Outline of behavioural interventions reported per UK specialty

	Education	Persuasion	Incentivise	Coercion	Training	Restriction	Environmental restructure	Modelling	Enablement	Communication	Guidelines	Fiscal	Regulation	Legislation	Environmental	Service provision
Endocrinology																
Geriatrics																
Psychiatry																
Emergency Medicine																
Neurology																
Cardiology																
Ophthalmology																
Paediatric surgery																
Paediatrics											1					
Anaesthetics																
Breast Surgery																
Vascular surgery																
Obstetrics & Gynaecology																
Intensive Care											1					
Neurosurgery								1								
Transplant Surgery																
Dermatology																1
Haematology																
Urology											1					1
Plastic Surgery																
Gastroenterology											1					
Respiratory																
Orthopaedics								1			1					
Rheumatology																
General Surgery (ASiT)	1						1		1				1			
Primary Care	2^a^	2^a^					1^a^	1	1		2^a^					
Nephrology									1		1					
Genitourinary Medicine																
General Surgery							2		1		2		1			1
Infection/Microbiology	3^a^	5^a^			1	1	5^a^		5	2^a^	6^a^		1		2^a^	8^a^

Legend: ^a^Interventions may have been part of a bundle of interventions reported in one abstract

NB. One behaviour change intervention has been excluded as the full nature of the intervention was not clearly defined

In total, 71 unique behaviour change functions were identified across the 56 abstracts reported behaviour change interventions (Table 2). Eight abstracts were deemed to describe multiple behaviour change interventions with six of these being reported at the infectious diseases/microbiology conference [29] and two at the primary care conference [38]. Policy categories; “guidelines” (16/71) and “service provision” (11/71) were the most frequently reported. Intervention functions; “education” (6/71), “persuasion” (7/71), “enablement” (9/71), and environmental restructuring (9/71) were also common. Intervention categories “incentivisation” and “coercion” and policy categories “fiscal” and “legislation” were not reported in any interventions. However, only infection/microbiology and primary care tended to report a broad variety of interventions, with the majority of interventions reported in the remaining ten specialties tending to focus on enablement (intervention) and guidelines or service provision (policy). The types of functions reported in abstracts that described multiple behaviour change interventions (8/56; 14%) are highlighted in Table 3. In abstracts reporting multiple behaviour change intervention functions there was a mix of policy and intervention targets with guidelines featuring in 6/8 (75%), environmental restructuring, education and persuasion all featuring in 4/8 (50%), and service provision in 3/8 (38%) of the abstracts.

**Table 3:** Outline of intervention functions reported in abstracts reporting multiple behaviour change interventions

Primary Care	1.	Guideline, persuasion, & modelling
	2.	Education, persuasion & environmental restructuring
Infection/Microbiology	3.	Guideline, persuasion & environmental
	4.	Guideline, persuasion & service provision
	5.	Guideline, environmental restructuring, education, communication
	6.	Guideline, education, service provision, environmental restructure
	7.	Guideline & service provision
	8.	Education & environmental restructuring

## Discussion

Clinical state-of-the-art conferences provide an opportunity for medical professionals to participate in research and reporting. They also allow us to gain an insight into different levels of research being undertaken within the field; from small scale research undertaken at the local level, to large scale studies being performed by key opinion leaders and organisations. This provides a window into the activities within specialties that is less influenced by publication bias than can often be observed through systematically reviewing peer-reviewed publications. Within this study, we observed a low rate of behaviour change intervention reporting across the majority of specialty state-of-the-art conferences in 2015. Infection specialties reported a significantly greater number and broader variety of AMS-AMR interventions with the majority of interventions reported by non-infection specialties falling into a narrow band of intervention and policy based functions.

These observations are concerning given the recent focus placed upon the need for cross-specialty engagement with AMS-AMR and behaviour change interventions. This has been supported broadly in the literature [4, 13, 16, 21, 22, 39]; by national organisations including Public Health England (PHE) [18], the British Society for Antimicrobial Chemotherapy (BSAC), and government [40]; and major international governments and organisations [6, 7]. These findings highlight the need to broaden the focus of AMS campaigns beyond infection specialties to promote leadership from within cohorts, which can drive behaviour change towards antimicrobial use.

This must be supported by clear and defined tools to help specialties engage and design AMS-AMR behaviour change interventions and assess the impact of these on patient outcomes. Whilst the use of behaviour change taxonomies allows content of interventions to be coded and categorised, facilitating the analysis of behaviour change interventions [24, 41], they are not appropriate for determining the effectiveness of interventions. Furthermore, for the field of AMS-AMR there remains no definition on what an appropriate behaviour change intervention is. This makes evaluating behaviour change challenging as certain functions of any framework used may be irrelevant or actually have a negative impact on behaviours within this context. [24, 41, 42] Kok and colleagues argue that as behavioural determinants are often specific to behaviours, populations, and contexts characterisations should thus be individualised and tailored for such [24, 41]. This will require engagement and drive from within clinical specialties to review current practices, define the context in which AMS-AMR interventions need to be implemented, and then tailor behaviour change interventions to optimise their effect within their local environment.

Finally, in a previous study “high risk” specialties were identified that currently use large amounts of antimicrobials and also experience high levels of healthcare associated infections [21]. This study reported that certain “high risk” specialties such as infection and intensive care had relatively high levels of engagement with AMS-AMR, whilst other specialties such as haematology and nephology tended to have a low apparent engagement at scientific conferences [21]. Within this study, we have observed an overall low rate of behaviour change interventions across all high risk specialties reported previously with only infection [29] and nephrology [43] reporting any behaviour change interventions in 2015. Furthermore, specialties with relatively high coverage of AMS-AMR at scientific conferences, such as Genitourinary Medicine, failed to report on behaviour change interventions despite having 9% coverage of AMS-AMR topics in 2015. This highlights the need for greater pan-specialty promotion of behaviour change interventions for AMS-AMR given the significant lack of focus on reporting such interventions currently.

There are several limitations to this study. Firstly, we only selected one leading state-of-the-art conference for each major clinical specialty in the UK. This makes our findings difficult to generalise across other countries and also may have introduced bias through excluding smaller, conferences and meetings, where specialties may have had more of an AMS-AMR focus. However, this method was selected as we aimed to generate a representative picture of current behaviour change interventions and the importance placed upon this by different specialties. By selecting leading state-of-the-art conferences we hoped that this would reflect the current overall importance of this subject within the specialty as well as allow for a more representative view of work being undertaken in the field. Secondly, we did not review invited talks and seminars provided by conferences given that they often were not presenting original data or results. Furthermore, as the taxonomy used to describe behaviour change does not allow for evaluation of the effectiveness of interventions it is not possible to evaluate whether interventions described were “appropriate” for the context in which they were described. Finally, as only a relatively small number of specialists from a specific geographical area were contacted to seek opinions on defining the largest UK conference in their field this may have introduced bias in our conference selection. To address this we ensured that two specialists from each field were contacted for their opinions. When there was discrepancy in responses from the individuals, conference attendance size was considered as the determinant with the conference with the largest attendance selected.

## Conclusion

In conclusion, despite evidence to support the role of behaviour change interventions for improving antimicrobial prescribing, very few studies reported implementing them at UK state-of-the-art conferences in 2015. Future research must focus on providing appropriate frameworks and mechanisms to allow clinical specialties to engage with AMS-AMR and design and evaluate the impact of behaviour change interventions within their specific contexts.

## Additional file

Additional file 1: Table S1. Behaviour change taxonomy used for classification of interventions reported in state-of-the-art scientific conference abstracts in 2015. (DOC 36 kb)
