# Supplementary material for: Behaviour change interventions to influence antimicrobial prescribing: a cross-sectional analysis of reports from UK state-of-the-art scientific conferences
Source: Antimicrob Resist Infect Control. 2017 Jan 13;6:11. doi: 10.1186/s13756-017-0170-7 (PMC5237267; doi:10.1186/s13756-017-0170-7)
Supplement: Additional file 1: Table S1. — Behaviour change taxonomy used for classification of interventions reported in state-of-the-art scientific conference abstracts in 2015. (DOC 36 kb) [file 13756_2017_170_MOESM1_ESM.doc]

**Additional file 1 Table S1**. Behaviour change taxonomy used for classification of interventions reported in state-of-the-art scientific conference abstracts in 2015

| **Intervention** |  |  |
| --- | --- | --- |
|  | Education | Increasing knowledge & understanding |
|  | Persuasion | Communication used to induce positive or negative feelings or drive actions |
|  | Incentivisation | Creating expectation of rewards for actions |
|  | Coercion | Creating expectation of punishment for actions |
|  | Training | Developing new skills |
|  | Restriction | Use of rules to reduce or increase the engagement in a target behaviour (whether positive or negative) |
|  | Environmental restructuring | Changes in the physical or social context |
|  | Modelling | Providing examples for people to aspire to / imitate |
|  | Enablement | Increasing means or reducing barriers to increase capability to achieve a goal or behaviour |
| **Policy** |  |  |
|  | Communication | Using print, electronic, telephonic, or broadcast media |
|  | Guidelines | Creating documents that recommend or mandate practice. |
|  | Fiscal | Taxing actions to reduce or increase a financial cost |
|  | Regulation | Establishing rules or principles of behaviour or practice |
|  | Legislation | Making or changing laws |
|  | Environmental | Designing or controlling the social environment |
|  | Service Provision | Delivering service |

**Legend:** Adapted from Michie et al. Implementation Science 2011, 6:42 <http://www.implementationscience.com/content/6/1/42>
